# Effects of Omega-3 Fatty Acid Supplementation on Cognitive Functions and Neural Substrates: A Voxel-Based Morphometry Study in Aged Mice

**DOI:** 10.3389/fnagi.2016.00038

**Published:** 2016-03-04

**Authors:** Debora Cutuli, Marco Pagani, Paola Caporali, Alberto Galbusera, Daniela Laricchiuta, Francesca Foti, Cristina Neri, Gianfranco Spalletta, Carlo Caltagirone, Laura Petrosini, Alessandro Gozzi

**Affiliations:** ^1^Santa Lucia FoundationRome, Italy; ^2^University of Rome “Sapienza”Rome, Italy; ^3^Functional Neuroimaging Laboratory, Istituto Italiano di TecnologiaRovereto, Italy; ^4^Center for Mind and Brain Sciences, University of TrentoRovereto, Italy; ^5^University of Rome “Tor Vergata”Rome, Italy

**Keywords:** aging, cognitive decline, omega-3 fatty acids, dietary supplementation, MRI, voxel-based morphometry

## Abstract

Human and experimental studies have revealed putative neuroprotective and pro-cognitive effects of omega-3 polyunsaturated fatty acids (n-3 PUFA) in aging, evidencing positive correlations between peripheral n-3 PUFA levels and regional grey matter (GM) volume, as well as negative correlations between dietary n-3 PUFA levels and cognitive deficits. We recently showed that n-3 PUFA supplemented aged mice exhibit better hippocampal-dependent mnesic functions, along with enhanced cellular plasticity and reduced neurodegeneration, thus supporting a role of n-3 PUFA supplementation in preventing cognitive decline during aging. To corroborate these initial results and develop new evidence on the effects of n-3 PUFA supplementation on brain substrates at macro-scale level, here we expanded behavioral analyses to the emotional domain (anxiety and coping skills), and carried out a fine-grained regional GM volumetric mapping by using high-resolution MRI-based voxel-based morphometry. The behavioral effects of 8 week n-3 PUFA supplementation were measured on cognitive (discriminative, spatial and social) and emotional (anxiety and coping) abilities of aged (19 month-old at the onset of study) C57B6/J mice. n-3 PUFA supplemented mice showed better mnesic performances as well as increased active coping skills. Importantly, these effects were associated with enlarged regional hippocampal, retrosplenial and prefrontal GM volumes, and with increased *post mortem* n-3 PUFA brain levels. These findings indicate that increased dietary n-3 PUFA intake in normal aging can improve fronto-hippocampal GM structure and function, an effect present also when the supplementation starts at late age. Our data are consistent with a protective role of n-3 PUFA supplementation in counteracting cognitive decline, emotional dysfunctions and brain atrophy.

## Introduction

The constant worldwide growth of the elderly population has amplified the interest in the prevention and improvement of age-related cognitive decline. Such a process is characterized by a progressive and irreversible loss of grey matter (GM) in many brain regions, with a prominent atrophy of the hippocampus and prefrontal lobes (Jernigan et al., 2001; Driscoll et al., 2006; Masliah et al., 2006). Research on environmental factors that affect age-related cognitive decline has aroused growing interest in cost-effective interventions, such as nutritional supplementation (Gómez-Pinilla, 2008; Maruszak et al., 2014).

As major components of neuronal membranes and key modulators of neuroinflammation, oxidative stress, and neurogenesis (Luchtman and Song, 2013; Denis et al., 2015), omega-3 polyunsaturated fatty acids (n-3 PUFA), particularly docosahexaenoic acid (DHA), eicosapentaenoic acid (EPA) and docosapentaenoic acid (DPA), may exert beneficial and neuroprotective effects on the aging brain. Consistently, rodent studies have shown that n-3 PUFA supplementation improves neurogenesis and synaptogenesis, executive functions and learning abilities, while n-3 PUFA deficiency is associated with memory deficits and impaired hippocampal plasticity (Fedorova and Salem, 2006; Hooijmans et al., 2012; Denis et al., 2013; Luchtman and Song, 2013; Maruszak et al., 2014). Human longitudinal studies based on direct or indirect indices of n-3 PUFA consumption have also provided evidence of n-3 PUFA beneficial role in aging. Namely, increased n-3 PUFA consumption correlates with better cognitive functioning and reduced risk for dementia (Beydoun et al., 2007; Dullemeijer et al., 2007; Whalley et al., 2008; Cunnane et al., 2009; Kröger et al., 2009; Samieri et al., 2011), higher total brain and regional GM volumes (Conklin et al., 2007; Samieri et al., 2012; Tan et al., 2012; Titova et al., 2013; Pottala et al., 2014) and reduced white matter (WM) hyperintensity (Bowman et al., 2013; Virtanen et al., 2013). However, interventional studies aimed at establishing a causative effect of n-3 PUFA supplementation on GM volumes and cognitive functions have produced inconclusive results. In fact, although some studies reported that n-3 PUFA supplementation improves cognition in healthy elderly subjects (Yurko-Mauro et al., 2010; Witte et al., 2014) and in patients with mild cognitive impairment (Chiu et al., 2008), other studies failed to reveal any significant effect in healthy subjects (van de Rest et al., 2008; Dangour et al., 2010; Geleijnse et al., 2012) and in patients with Alzheimer’s disease (AD; Quinn et al., 2010). Uncontrolled confounding factors, such as socio-economic status, genetic background as well as healthy habits and lifestyle (e.g., exercise, not smoking, sleep, social support, use of vitamin supplement, etc.) may contribute to these inconsistent results and make it difficult to isolate the specific neuroprotective impact of n-3 PUFA-enriched diet on cognitive functions of elderly people(Denis et al., 2013; Raji et al., 2014). Furthermore, the enormousvariation in n-3 PUFA supplement kind and dosage, and a general failure in controlling for n-6 PUFA dietary intake may also account for the huge variability in human clinical and interventional studies.

As a result, the impact of n-3 PUFA supplementation on cognitive functions in the aging human brain and the underlying neuronal substrates is still a matter of debate. Studies under controlled environmental and genetic conditions in animal models can help to disambiguate the complex relationships among n-3 PUFA intake, cognitive performance and GM morphometry. We recently demonstrated that 8-week n-3 PUFA supplementation in aged mice robustly ameliorates hippocampal functions via increased neurogenesis and reduced neurodegenerative processes (Cutuli et al., 2014). However, whether cellular-scale hippocampal changes can result in macro-scale structural alterations detectable through volumetric MRI, and whether n-3 PUFA effects are limited to hippocampal areas or affect other neocortical and/or subcortical regions remain to be determined.

In order to address these issues, in the present study MRI volumetric measures of the entire brain (and not just of hippocampal regions) as well as cognitive and emotional functions not previously evaluated were assessed. To this aim in aged inbred mice undergoing n-3 PUFA supplementation with respect to isocaloric control conditions we measured cognitive abilities in different spatial and discriminative tasks, and in tasks assessing sociability and social memory. Age-related disorders are in fact reported to affect social memory abilities (Riedel et al., 2009). Notably, since in older populations cognitive decline is frequently associated with depressive-like symptoms (Steffens et al., 2006) and n-3 PUFA are reported to exert antidepressant action (Freeman et al., 2006; Sublette et al., 2011), the behavioral assessment was extended to emotional competencies in facing coping skills. A control of anxiety levels was also performed. Then, regional GM volumes were mapped by using high resolution MRI-based whole-brain voxel-based morphometry (VBM) (Dodero et al., 2013; Sannino et al., 2015). Finally, *ex vivo* brain levels of n-3 PUFA and individual behavioral scores were correlated with regional GM volumes to assess whether n-3 PUFA levels can be considered reliable predictors of volume changes and behavioral outcomes.

## Materials and Methods

### Animals

**FIGURE 1:**
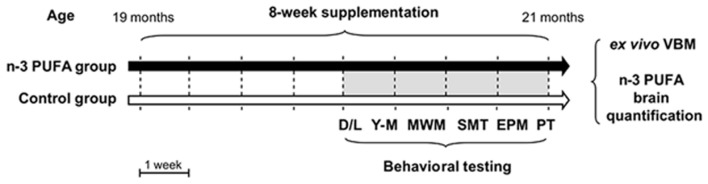
**Global timing of the experimental procedure.** Experimental groups of aged mice (n-3 PUFA and controls), dietary supplementation duration (8 weeks), behavioral testing (D/L, dark/light test; Y-M, Y-maze test with objects; MWM, Morris Water Maze; SMT, sociability and social memory test; EPM, Elevated Plus Maze; PT, Porsolt test) and *ex vivo* neuroimaging (VBM) and metabolic analyses (n-3 PUFA brain levels) are indicated.

Male aged C57B6/J mice (19 month-old at the onset of study; 35.57 ± 0.69 g) were used in the present research (Charles River Laboratories, Italy). The animals were group-housed (three-four mice/cage) with temperature (22–23°C) and humidity controlled (60 ± 5%), under a 12:12 h light/dark cycle with free access to food and water. Animals were randomly divided in two groups: (1) mice supplemented with an n-3 PUFA mixture by daily gavage for 8 weeks (5 day/week) (Group name: n-3 PUFA; *n* = 11); (2) mice supplemented with olive oil by daily gavage for the same period used as controls of an isocaloric intake, as reported in previous studies (Kotani et al., 2006; Oarada et al., 2008; Nakamoto et al., 2010; Sinn et al., 2010; Danthiir et al., 2011; Vinot et al., 2011; Cutuli et al., 2014) (Group name: Control; *n* = 10) (**Figure 1**). Animals’ weight was recorded weekly throughout the study. No significant differences between groups were found in mice body weight during the entire experimental period [two-way ANOVA (group × week): group: *F*_1,19_ = 0.32, *p* = 0.58; week: *F*_9,171_ = 3.57, *p* = 0.0004; interaction: *F*_9,171_ = 0.47, *p* = 0.89].

### Food Supplementation

Food supplementation was performed by daily gavage to ensure that all cagemates received the same controlled amount of dietary supplements regardless of social hierarchy or appetitive drive.

n-3 PUFA group was supplemented with a volume of 0.015 ml of fatty acids mixture (Pfizer, Italy) corresponding to a dose of 360 mg/kg/day of n-3 PUFA (Calviello et al., 1997; Cutuli et al., 2014) mainly constituted by EPA (20:5 n-3; 63%), DHA (22:6 n-3; 26%), DPA (22:5 n-3; 4%), and α-linolenic acid (ALA, 18:3 n-3; 1%) (Cutuli et al., 2014). Control group was supplemented with the same volume of olive oil (Trasimeno, Italy) containing ≈ 4 mg/kg/day of n-3 PUFA constituted only by ALA (1%) (Cutuli et al., 2014). The two groups of animals were fed *ad libitum* with standard food pellets (Mucedola 4RF21 standard diet GLP complete feed for mice and rats; Mucedola, Italy).

### Experimental Procedures

Starting from the fifth supplementation week (**Figure 1**), mice were tested in a number of behavioral tasks tapping distinct cognitive and emotional functions: Dark/Light test, Y-maze test with objects, Morris Water Maze (MWM), Sociability and Social Memory test (SMT), Elevated Plus Maze (EPM), and lastly Porsolt test. After behavioral testing, the animals were sacrificed to perform VBM and biochemical analyses.

### Behavioral Testing

#### Dark/Light Test

At the beginning of behavioral testing, anxiety levels and exploratory behaviors were tested by means of the Dark/Light test that is based on the innate rodent tendency to avoid brightly illuminated areas and to spontaneously explore novel environment (Crawley and Goodwin, 1980). Dark/Light test was performed in an apparatus consisting of a parallelepiped box containing two compartments: a dark compartment (18 cm × 42 cm × 21 cm) with black walls, and a lighted compartment (36 cm × 42 cm × cm 21 cm) with white walls. The two compartments were separated by a wall pierced with an open door (7 cm × 7 cm). The mouse was placed in the lighted compartment facing the wall and allowed to freely explore both compartments for 10 min.

The following parameters were analyzed: time spent in each compartment; latency of first entrance into the dark compartment; number of transitions to the dark compartment; number of defecations.

#### Y-Maze Test with Objects

To assess novel object recognition memory we used a Y-Maze test with objects (Winters and Bussey, 2005). The Y-Maze apparatus was made of gray Plexiglas and consisted of three identical arms (8 cm × 30 cm × 15 cm) with a 120° angle between adjacent arms. The three arms were designated as: start arm, from which the mouse started to explore the maze, and two choice arms, containing or not objects. Y-Maze test with objects was performed in a dimly lighted room and consisted of three trials. During the first trial (habituation phase) lasting 5 min the mice placed in the start arm were allowed to freely explore the Y-Maze arms containing no objects. After 3 min-inter-trial interval (ITI) the mice underwent the second trial (training phase) lasting 5 min during which moving from the start arm they were allowed to explore two identical objects put at the end of the choice arms. After 1 h-ITI the mice underwent the last 5 min-trial (retention phase) during which they were allowed to freely explore one copy of the familiar object and a novel object put at the end of the choice arms. During the ITI mice were put in their homecages. Maze floor and walls were cleaned after each trial to remove olfactory cues. Trials were recorded by a ceiling-mounted camera and analyzed by a video analyser (Ethovision XT, Noldus, The Netherlands).

To evaluate the preference for the novel object (novelty) total time spent in contact with the familiar *vs.* novel object during the retention phase was analyzed. The discrimination index was calculated: contact time with the novel object (T_no_) *minus* contact time with the familiar one (T_fo_)/total contact time with both objects.

#### Morris Water Maze

Morris Water Maze test is a well validated test to assess localizatory abilities in rodents during aging (Chen et al., 2000; Carrié et al., 2002). The mice were placed in a circular white pool (diameter 140 cm) filled with 24°C water made opaque by the addition of atoxic acrylic white color (Giotto, Italy) (Cutuli et al., 2014). An escape platform (diameter 5 cm) with a rough surface was placed in the middle of the NW quadrant 20 cm from the side walls. It was submerged 0.5 cm under the water level. The pool located in a room uniformly lighted by four lamps (25 W each) was surrounded by several extra-maze cues. The water maze was surmounted by a video camera whose signal was relayed to a monitor and to the image analyser (Ethovision XT, Noldus, The Netherlands). The protocol consisted of a 16-trial Place phase and a 1-trial Probe phase. During the Place phase, mice were submitted to four consecutive sessions made by four 90 s-trials per day. During the 15 min-inter-trial interval (ITI) mice were put in their home cages. At the beginning of each trial, mice were gently released into the water from pseudo-randomly varied starting points and were allowed to swim around to find the hidden platform. Mice that did not locate the platform within 90 s were gently guided there by the experimenter. After mice climbed the platform, they were allowed to remain on it for 30 s. After 24 h, mice were submitted to the Probe phase, in which the platform was removed and the mice were allowed to search for it for 60 s.

To evaluate localizatory memory the following MWM parameters were analyzed: time spent and distance swum to reach the platform during the Place phase; distance swum in the previously rewarded quadrant during the Probe phase. The navigational strategies were classified in three main categories, regardless of whether the platform was reached or not: Circling (C): circular swimming with inversion of direction and counter-clockwise and clockwise turnings; Searching (S): swimming around the pool in all or some quadrants, visiting the same area more than once; direct Finding (F): swimming toward the platform without any foraging around the pool. Two researchers unaware of the individual specimen’s group categorized the swimming trajectories drawn by the image analyzer. They attributed the dominant behavior in each trial to a specific category. Categorization was considered reliable only when their judgments were consistent.

#### Sociability and Social Memory Test

Sociability and SMT assesses social motivation and interest in social novelty, respectively (Nadler et al., 2004; Riedel et al., 2009; Cutuli et al., 2013). Rodents normally prefer to spend more time with another rodent (sociability) and investigate a novel intruder more than a familiar mouse (social novelty). Age-related disorders are reported to affect social memory abilities (Riedel et al., 2009). The apparatus consisted of a white rectangular wooden box (54 cm × 42 cm × 21 cm) divided in three 18 cm-wide chambers by two transparent Plexiglas walls with an open middle door (3.5 cm × 3.5 cm). Each lateral chamber contained a small metal cage (9 cm × 8 cm) with mesh-like holes in which stranger mice were confined for social interactions. The test comprised three trials: habituation, Sociability and SMT. During the habituation trial, the mice were allowed to freely move in the apparatus for 5 min. During Sociability trial, an unfamiliar juvenile (35–45 pnd) mouse conspecific (Stranger 1) was placed inside the small metal cage in one of the lateral chambers (randomly selected and counterbalanced for each group). The experimental mouse was placed in the apparatus and it was allowed to freely explore the three chambers and contact the small metal cages for 5 min. During SMT, another unfamiliar juvenile mouse (Stranger 2) was placed inside the metal cage in the opposite lateral chamber that was empty during the Sociability session. The experimental mouse was allowed to freely move and contact the metal cages for 5 min. ITI between habituation and Sociability trials lasted 3 min, while ITI between Sociability trial and SMT lasted 1 h. Mice behavior was recorded by a video camera mounted on the ceiling. The resulting video signal was relayed to a monitor and to an image analyzer (Ethovision XT, Noldus, The Netherlands).

Time spent in each lateral chamber during Sociability and SMT was recorded. Discrimination indexes were calculated: sociability index = contact time with the Stranger 1 (T_S1_) *minus* contact time with the empty cage (T_e_)/total contact time; social memory index = contact time with the Stranger 2 (T_S2_) *minus* contact time with the Stranger 1 (T_S1_)/total contact time.

#### Elevated Plus Maze

Elevated Plus Maze is a well validated test to assess anxiety levels in rodents based on their natural aversion to open spaces (Ruehle et al., 2013; Cutuli et al., 2014). The maze was formed by a wooden structure in the shape of a cross with a central platform and four 35 cm × 6 cm arms raised 100 cm above the ground. The north and south arms were open, the east and west arms were enclosed by walls 20 cm high. During a 5-min trial the mouse was placed in the central platform and allowed to freely explore the maze. Since mice avoid open areas by confining movements to enclosed spaces or to the edges of a bounded space, a typical mouse tends to spend the majority of trial time in the closed arms. The entire apparatus was cleaned after each trial to remove olfactory cues. Trials were recorded by a ceiling-mounted camera and analyzed by an image analyzer (Ethovision XT, Noldus, The Netherlands).

The following EPM parameters were measured: total entries and total time spent in the open and closed arms; number of defecations.

#### Porsolt Test

Mice were gently placed in individual glass cylinders (height 40 cm; diameter 18 cm) containing 20 cm water at 28 ± 2°C. Mice were exposed to the apparatus for 10 min. At the end of the test mice were removed from the cylinder, allowed to dry in a small cage placed under a heat source and returned to their homecages. The behavior exhibited by each animal during the test was recorded by using a frontally mounted camera. Later, an observer blind to the treatment received by each animal manually scored the videos (Ethovison XT, Noldus, The Netherlands).

Duration and frequency of the following behaviors were taken into account (Keers et al., 2012; Costa et al., 2013):

– passive behaviors: immobility = total absence of movement; paddling = small movements of one of the posterior paws not producing displacement;

– active behaviors: swimming = large and horizontal movements of the paws leading to displacement of the body around the cylinder; climbing = vigorous vertical movements of the forepaws, directed against the wall of the tank, leading to displacement the body around the cylinder.

### Sample Preparation and MR Acquisition

High-resolution morpho-anatomical T2-weighted MR imaging of *ex vivo* mouse brains was performed in paraformaldehyde (4% PFA; 100 ml) fixed specimens. Standard sample preparation and MRI acquisition have been recently described (Dodero et al., 2013). Briefly, C57Bl/6 mice supplemented with n-3 PUFA (all 87-week-old) and age-matched controls supplemented with olive oil were deeply anesthetized with an intraperitoneal avertin injection (375 mg/kg) and their brains were perfused *in situ* via cardiac perfusion. The perfusion was performed with phosphate buffered saline followed by paraformaldehyde (4% PFA; 100 ml). Both perfusion solutions were added with a Gadolinium chelate (Prohance, Bracco, Milan) at a concentration of 10 and 5 mM, respectively, to shorten longitudinal relaxation times. Brains were imaged inside intact skulls to avoid post-extraction deformations. A multi-channel 7.0 Tesla MRI scanner (Bruker Biospin, Milan) was used to acquire anatomical images of the brain, using a 72 mm birdcage transmit coil, a custom-built saddle-shaped solenoid coil for signal reception, and the following imaging parameters: FLASH 3D sequence with TR = 17 ms, TE = 10 ms, α = 30°, matrix size of 260 × 180 × 180, field of view of 1.83 cm × 1.26 cm × 1.26 cm and voxel size of 0.07 mm (isotropic).

### Image Processing and Analysis

Inter-group morpho-anatomical differences in local GM volumes were mapped with VBM (Ashburner and Friston, 2000) using ANTs (Avants et al., 2009). Registration-based VBM procedure on the mouse brain has been thoroughly described (Pagani et al., 2012) and is briefly reported herein to provide a comprehensive description of all the experimental procedures involved.

First, all the high-resolution T2W images were corrected for intensity non-uniformity and skull stripped to remove extra-brain tissue. Second, a study-based template was created aligning pre-processed images to a common reference space using affine and diffeomorphic registrations. Third, individual images of the two groups were registered to the study-based template using affine and diffeomorphic registrations. Fourth, spatially normalized images were segmented to calculate tissue probability maps using Markov Random Field to enforce the spatial smoothing of the tissues. The separation of the different tissues is improved by initializing the process with the probability maps of the study based template previously segmented. Fifth, the Jacobian determinants of the deformation field were extracted and applied to modulate the GM probability maps calculated during the segmentation. This procedure allowed the analysis of GM probability maps in terms of local volumetric variation instead of tissue density. Jacobian determinants were also normalized by the total intracranial volume to further eliminate overall brain volume variations. Sixth, the resulting modulated GM probability maps were smoothed using a Gaussian kernel with a sigma of three voxel width and employed for voxel-wise statistics and thresholded with a cluster-based procedure as implemented in FSL.

Regional GM volume differences between n-3 PUFA and olive oil supplemented mice were mapped using a two-sample *t*-test (*p* < 0.01) followed by a cluster correction using a significant cluster threshold of *p* = 0.01 (Worsley et al., 1992). To ensure inter-group differences were not due to segmentation-artifacts reflecting indirect alterations in GM intensity, we performed tensor based morphometry (TBM) on the same subjects. TBM is a procedure that does not require tissue segmentation and can be used to map inter-group differences in local brain volume independent of the nature of the tissue quantified. The pre-processing steps employed for TBM are the same of VBM, with the exception of the segmentation, which is not performed.

To assess the correlations among the regional GM volumes, n-3 PUFA level and behavioral performances, we also performed voxel-wise Pearson’s correlation mapping by using individual n-3 PUFA brain concentration levels and behavioral scores as regressors (*p* < 0.05, followed by cluster level significance correction with a threshold of *p* = 0.01).

To explicit the correlative relationship between variables obtained in univariate maps, mean GM volumes were quantified in representative bilateral cubic (9 × 9 × 9 voxels) regions of interest (ROIs) centered in hippocampal foci exhibiting inter-group differences or areas of significant correlation. The exact anatomical location of the hippocampal ROIs selected for correlations is shown in Supplementary Figure S1.

### n-3 PUFA Quantification by GC/MS

After imaging, the brain content of n-3 PUFA was quantified. Fatty acids were extracted using the method reported by Folch et al. (1957) with slight modifications. Briefly, brains were homogenized in CHCl_3_/MeOH (2:1 v/v) to a final dilution of 20-fold of the original sample volume, assuming that the tissue has the same specific gravity of water. Heptadecanoic acid was used as internal standard. The resulting organic phase was evaporated to dryness in a speed-vac at room temperature and then derivatized with BSTFA-TMCS 99:1 v/v (Sigma–Aldrich, Italy) for 1 h at 60°C. Derivatized samples were transferred in the injection vial and added with 20% v/v of Acetone. GC/MS analyses were performed using a Focus GC (Thermo Scientific, USA) equipped with 30 m × 0.25 mm fused silica capillary column SLB^TM^-5MS (Supelco) and connected to a PolarisQ mass spectrometer (Thermo Scientific, USA). Two micro liter of samples were injected in split mode (1:10 ratio), the injector temperature was set at 200°C; the carrier gas was Helium and the flow rate was maintained constant at 1 ml/minute. The initial oven temperature of 100°C was held for 1 min and then raised to 250°C at 10°C/minute and maintained for 6 min. After then the oven temperature was increased up to 310°C at 20°C/min and held for 5 min. Mass transfer line was maintained at 280°C and the ion source at 200°C. Analyses were performed in Selected Ion Monitoring (SIM) mode and fatty acids were identified by comparison with commercial standards.

### Statistical Analyses

All data were tested for normality (Shapiro–Wilk’s test) and homoscedasticity (Levene’s test) and presented as mean ± SEM. Behavioral data and biochemical correlates were analyzed by using one- and two-way ANOVAs (with group as between-factor and compartment/session/strategy/arm/behavior as within-factors) followed by Tukey’s HSD tests. Values of *p* < 0.05 were considered significant (Statistica 7, Statsoft).

### Ethical Statement

All experimental procedures were performed in accordance with the Italian law (D.L. 116, 1992 Italian Ministry of Health, Rome), and in agreement with the European Union Directive (2010/63/EU). All surgical procedures were performed under deep anesthesia and all efforts were made to minimize suffering and reduce the number of animals that were used. All experimental procedures were approved by the Italian Ministry of Health (Ministerial Decree number 232/2012-B, 10-2012).

## Results

### n-3 PUFA Supplemented Mice Exhibit Improved Mnesic Functions

**FIGURE 2:**
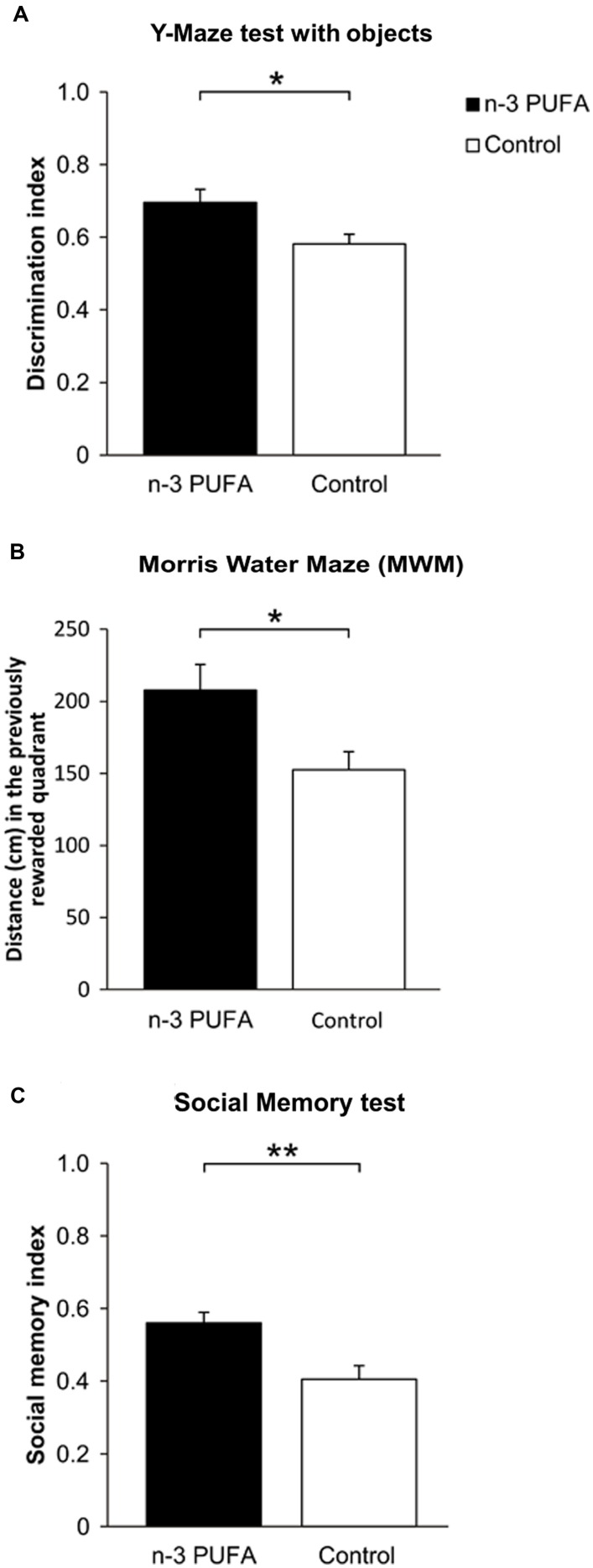
**n-3 PUFA supplementation effects on mnesic performances.** **(A)** Discrimination index in Y-Maze test with objects. **(B)** Distance (cm) swum in the previously rewarded quadrant during Probe phase of MWM. **(C)** Social memory index in SMT. Asterisks inside the graphs indicate the significance levels of comparisons between groups: ^∗^*p* < 0.05, ^∗∗^*p* ≤ 0.01.

To verify the ability of n-3 PUFA supplementation to improve mnesic function in the aged brain, both experimental aged mice groups were submitted to a battery of behavioral tests measuring hippocampal-dependent cognitive abilities (**Figure 2**). n-3 PUFA supplemented mice demonstrated better novelty recognition abilities in the Y-Maze test with objects (*F*_1,19_ = 6.13, *p* = 0.02, **Figure 2A**) as well as in the MWM (**Figure 2B**). In the latter test, no inter-group differences were observed during Place in latency (group: *F*_1,19_ = 0.52, *p* = 0.48; session: *F*_3,57_ = 4.85, *p* = 0.004; interaction: *F*_3,57_ = 0.74, *p* = 0.53) and distance swum (group: *F*_1,19_ = 0.17, *p* = 0.69; session: *F*_3,57_ = 17.23, *p* < 0.000001; interaction: *F*_3,57_ = 0.30, *p* = 0.82) to reach the hidden platform. No differences were also observed in navigational strategies (group: *F*_1,19_ = 1.55, *p* = 0.22; strategy: *F*_2,38_ = 310.94, *p* < 0.000001; interaction: *F*_2,38_ = 1.66, *p* = 0.20), with Searching as the most used strategy (*p* = 0.0001). However, during Probe phase n-3 PUFA mice exhibited higher platform location retention as measured by distance swum in the previously rewarded (platform) quadrant (*F*_1,19_ = 6.14, *p* = 0.02), thus supporting a beneficial effect of n-3 PUFA supplementation on hippocampal mnesic functions. Accordingly, while all mice displayed an equal preference for social stimuli (sociability index: *F*_1,19_ = 0.41, *p* = 0.53), n-3 PUFA supplementation significantly improved mnesic performances in SMT as indicated by the increased social memory index observed in n-3 PUFA supplemented mice (*F*_1,19_ = 10.88, *p* = 0.004; **Figure 2C**).

### n-3 PUFA Supplemented Mice Exhibit Improved Coping Skills and Unmodified Anxiety Levels

**FIGURE 3:**
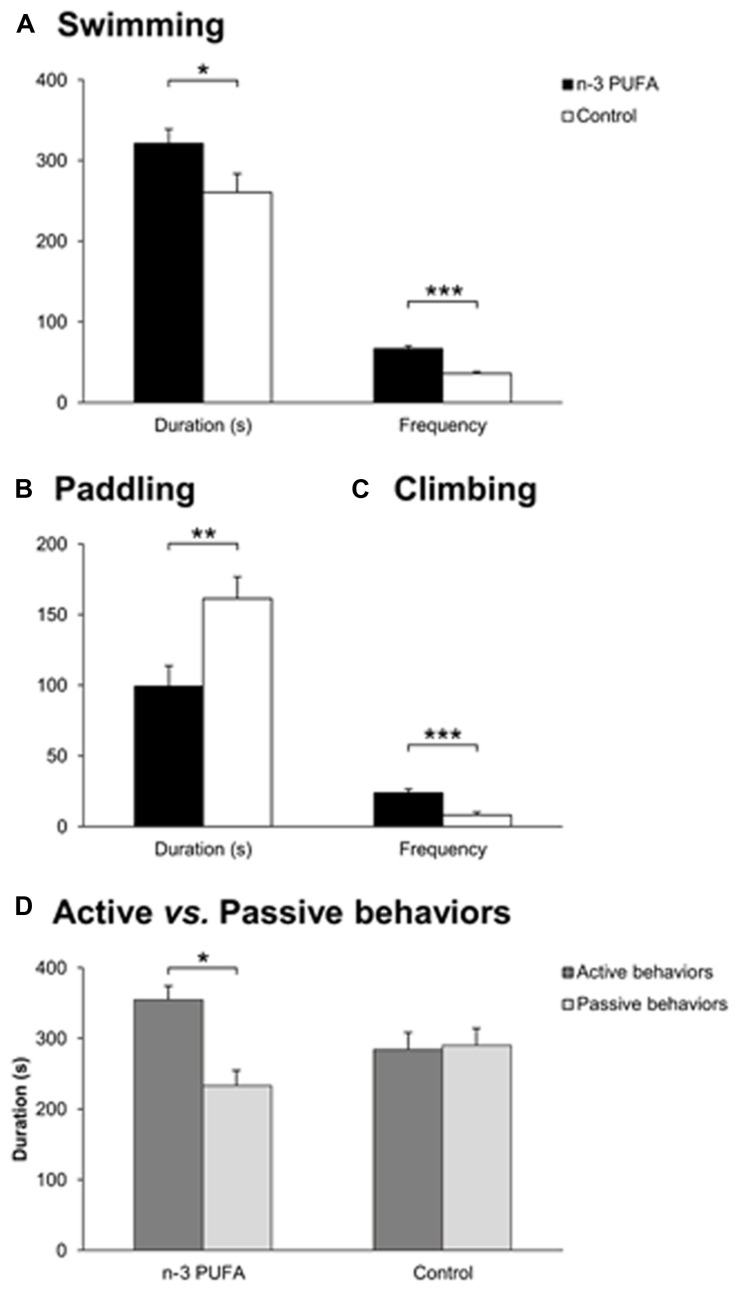
**n-3 PUFA supplementation effects on coping skills.** **(A)** Swimming duration (s) and frequency in Porsolt test. **(B)** Paddling duration (s). **(C)** Climbing frequency. **(D)** Active *vs.* passive behavior duration (s). Asterisks inside the graphs indicate the significance levels of comparisons between groups: ^∗^*p* < 0.05, ^∗∗^*p* ≤ 0.01, or ^∗∗∗^*p* ≤ 0.001.

As reduced hippocampal volumes, depression and cognitive deterioration are frequently associated in older populations (Videbech and Ravnkilde, 2004; Steffens et al., 2006), Porsolt test was used to assess depressive-like behaviors and coping strategies in the two experimental groups (**Figure 3**). Depressive-like traits appeared to be less prominent in n-3 PUFA supplemented mice with respect to controls as evidenced by higher duration (*F*_1,19_ = 4.69, *p* = 0.04) and frequency of swimming (*F*_1,19_ = 45.10, *p* < 0.000001), as well as higher frequency of climbing (*F*_1,19_ = 19.55, *p* = 0.0003) and lower duration of paddling (*F*_1,19_ = 8.61, *p* = 0.008) (**Figures 3A,C**). No treatment differences were observed for the remaining parameters (immobility, duration: *F*_1,19_ = 0.37, *p* = 0.55; frequency: *F*_1,19_ = 2.42, *p* = 0.14; paddling, frequency: *F*_1,19_ = 3.01, *p* = 0.09; climbing, duration: *F*_1,19_ = 1.47, *p* = 0.24). Interestingly, ANOVA performed on active *vs.* passive behaviors (group: *F*_1,19_ = 1.33, *p* = 0.26; behavior: *F*_1,19_ = 3.53, *p* = 0.08; interaction: *F*_1,19_ = 4.68, *p* = 0.04) revealed that the total duration of active behaviors (swimming + climbing) was significantly higher than the total duration of passive behaviors (immobility + paddling) in n-3 PUFA supplemented mice than in controls (*p* = 0.04) (**Figure 3D**), indicating increased use of active coping strategies in n-3 PUFA supplemented mice.

Importantly, no inter-group differences in terms of anxiety levels and explorative behaviors were observed. In fact, in the Dark/Light test all mice spent more time in the dark compartment than in the lighted one (group: *F*_1,19_ = 0.09, *p* = 0.76; compartment: *F*_1,19_ = 9.26, *p* = 0.007; interaction: *F*_1,19_ = 0.98, *p* = 0.33), without differences in the latency of first entrance (*F*_1,19_ = 1.87, *p* = 0.19), number of transitions into the dark compartment (*F*_1,19_ = 0.53, *p* = 0.48), and total defecations (*F*_1,19_ = 0.07, *p* = 0.79). These findings were confirmed also in the EPM test during which all mice spent significantly more time in the closed than open arms (group: *F*_1,19_ = 0.05, *p* = 0.82; arm: *F*_1,19_ = 1450.51, *p* < 0.000001; interaction: *F*_1,19_ = 0.12, *p* = 0.73), showing similar number of total entries (*F*_1,19_ = 0.03, *p* = 0.87) and no differences in defecation number (*F*_1,19_ = 2.59, *p* = 0.12).

### n-3 PUFA Supplemented Mice Exhibit Foci of Increased Hippocampal and Cortical GM Volume

**FIGURE 4:**
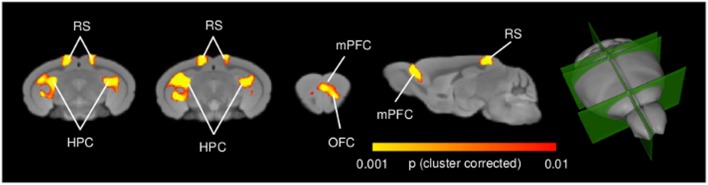
**n-3 PUFA supplementation increases hippocampal and prefrontal GM volume.** VBM morphometric analysis revealed significantly increased (*p* < 0.01, cluster corrected at a significance level of 0.01) regional GM volume in hippocampal formation, prefrontal and retrosplenial cortex in n-3 PUFA supplemented compared to control mice. 3D rendering of the sagittal and coronal slices is also visualized. HPC, hippocampus; mPFC, medial prefrontal cortex; RS, retrosplenial cortex.

High-resolution voxel-wise VBM mapping revealed prominent bilateral areas of increased GM volume in the posterior hippocampus, plus additional foci of GM increase in the retrosplenial and medial prefrontal cortices in n-3 PUFA supplemented mice compared to controls (*p* < 0.01, cluster-based correction, **Figure 4**). No foci of significant GM volume reduction were observed throughout the brain in n-3 PUFA supplemented mice (*p* < 0.01, cluster-based correction). TBM of n-3 PUFA and control groups produced similar inter-group difference maps with clear involvement of foci of hippocampal, retrosplenial and prefrontal areas (*p* < 0.01, cluster-based correction Supplementary Figure S2). The presence of analogous findings in VBM and TBM maps corroborates a supplementation-specific impact on the GM volumes mapped with VBM.

### Cognitive and Behavioral Performances Positively Correlate with Fronto-Hippocampal GM Volume

**FIGURE 5:**
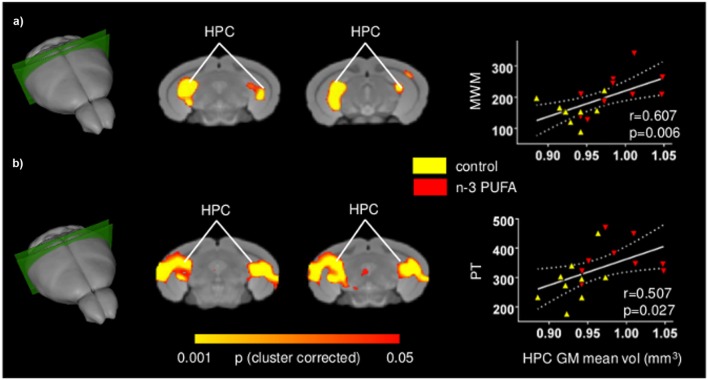
**Behavioral performances are positively correlated with hippocampal GM increase.** Voxel-wise correlation mapping of behavioral scores and GM volume. Foci of correlation between GM volume and enhanced MWM spatial mnesic performances **(a)** and increased coping skills in the Porsolt test **(b)** were found in the same regions exhibiting univariate increased GM volume. Scatter plots indicate significant Pearson’s correlations between n-3 PUFA concentrations and hippocampal mean GM volume (continuous lines), and the 95% CI (dotted lines). Red triangles indicate n-3 PUFA supplemented subjects, yellow triangles indicate the control group subjects. HPC, hippocampus; MWM, Morris Water Maze; PT, Porsolt test.

In an attempt to probe the relationship between cognitive and behavioral performance and regional GM volumes, and locate the brain substrates underlying the different performance levels of the two groups, voxel-wise regression of individual behavioral parameters was performed on GM maps at the subject level. **Figure 5** depicts significant voxel-wise correlation mapping obtained by using individual behavioral scores as regressors. Consistent with univariate mapping of supplementation effects, foci of significant voxel-wise correlations were observed in hippocampal formation when behavioral scores from MWM and Porsolt test were used (*p* < 0.05, cluster-based correction). In good agreement with univariate inter-group maps, additional foci of significant correlation were found in retrosplenial and medial prefrontal cortices, an effect that, however, did not survive cluster-based correction (Supplementary Figure S3). Overall, these findings support the involvement of the mapped GM substrates in the improved cognitive and increased coping skills exhibited by n-3 PUFA supplemented mice. No significant correlations were found for any of the remaining behavioral variables mapped.

### n-3 PUFA Supplemented Mice Exhibit Increased EPA and DHA Brain Levels

**FIGURE 6:**
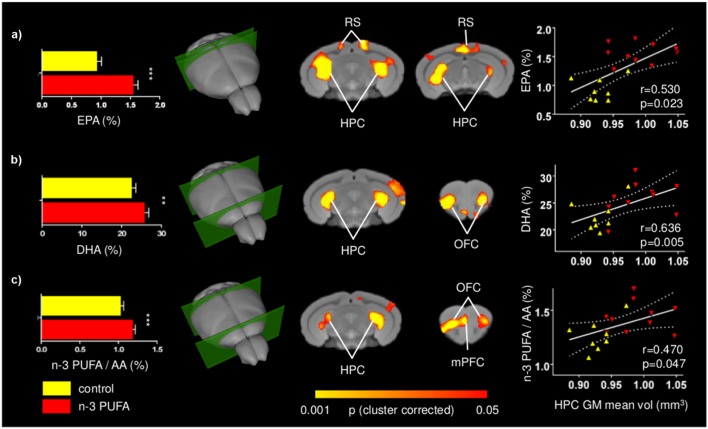
**Positive correlations between n-3 PUFA levels and fronto-hippocampal GM volume.** Voxel-wise correlation mapping of total brain EPA **(a)**, DHA **(b)** and n-3 PUFA/AA concentrations **(c)** and GM volume revealed foci of significant correlation in the same regions exhibiting univariate increased GM (hippocampal, prefrontal, retrosplenial, and orbitofrontal areas). Histograms report mean concentrations with error bars indicating SEM. Scatter plots indicate significant Pearson’s correlations between n-3 PUFA concentrations and hippocampal mean GM volume (continuous lines), and the 95% CI (dotted lines). Red triangles indicate n-3 PUFA supplemented subjects, yellow triangles indicate the control group subjects. HPC, hippocampus; mPFC, medial prefrontal cortex; RS, retrosplenial cortex; OFC, orbitofrontal cortex; EPA, eicosapentaenoic acid; DHA, docosahexaenoic acid; AA, arachidonic acid. Asterisks inside the graphs indicate the significance levels of comparisons between groups: ^∗∗^*p* ≤ 0.01, or ^∗∗∗^*p* ≤ 0.001.

To assess effectiveness of the supplementation regime, the concentrations of EPA, DHA, and DPA, the three major n-3 PUFA components of cell membranes, were measured. EPA+DHA+DPA/Arachidonic Acid (AA) ratio was also measured given its postulated role in cognitive dysfunction and neuroinflammation (Rao et al., 2007; Labrousse et al., 2012). EPA and DHA, but not DPA, levels were found to be increased in the n-3 PUFA group in comparison to controls, as revealed by one-way ANOVAs (EPA: *F*_1,19_ = 68.36, *p* < 0.000001; DHA: *F*_1,19_ = 7.11, *p* = 0.01; DPA: *F*_1,19_ = 0.10, *p* = 0.75). Moreover, one-way ANOVA on the EPA+DHA+DPA/AA ratio revealed a significant increase of EPA + DHA + DPA/AA ratio in n-3 PUFA group in comparison to controls (*F*_1,19_ = 7.55, *p* = 0.01) (**Figure 6**).

### n-3 PUFA Brain Levels Positively Correlate with Fronto-Hippocampal GM Volume

To further probe the relationship between n-3 PUFA supplementation and GM morphometric alterations mapped, voxel-wise correlations of n-3 PUFA levels of individual subjects were generated. Voxel-wise correlation of total EPA, DHA and n-3 PUFA/AA concentrations revealed foci of significant correlations in the hippocampal, retrosplenial and prefrontal regions, as well as orbitofrontal areas (*p* < 0.05, cluster-based correction; **Figure 6**). Additional foci of significant voxel-wise correlations between GM volumes and DHA levels were found in the medial prefrontal cortex in uncorrected statistics maps (Supplementary Figure S4). The anatomical location of these correlations is consistent with the effects of n-3 PUFA supplementation on GM volume and behavioral performance changes.

## Discussion

As main components of synaptic membranes, n-3 PUFA have an important role in keeping structure and function of aged brain, a feature that has promoted research on their dietary supplementation as a strategy to counteract aging-related cognitive decline. However, despite encouraging epidemiological evidence linking enhanced peripheral n-3 PUFA levels to improved cognitive performance and brain structure (Denis et al., 2015), interventional studies on n-3 PUFA supplementation have so far produced inconsistent results. This issue could reflect methodological inconsistencies such as the contribution of genetic and environmental factors that cannot be effectively controlled in human studies. In the present work we sought to overcome these limitations by investigating the relationships between GM volumes, cognitive and emotional performances, and n-3 PUFA cerebral levels in genetically homogeneous inbred aged mice reared in controlled laboratory conditions. In particular, we investigated whether long-term n-3 PUFA supplementation starting at old age may produce behavioral improvements and how the eventual improvements can be related to underlying neuroanatomical substrates. The present results strongly corroborate the emerging view of a pro-cognitive and neuroprotective function of n-3 PUFA supplementation on the aged brain (Denis et al., 2013; Varteresian and Lavretsky, 2014). Specifically, n-3 PUFA supplemented mice exhibited improved mnesic functions and coping skills, and presented foci of greater GM volumes in fronto-hippocampal areas. The increased GM volumes correlated with better mnesic performances, increased active coping skills, and enhanced total brain n-3 PUFA concentrations. Collectively, these findings indicate that the n-3 PUFA-induced neuroprotective effects are able to take place even when the supplementation starts at late age. Importantly, the present results were obtained through commonly available supplements (i.e., commercially available n-3 PUFA mixture and olive oil) and employing a well-established brain structural VBM readout to maximize ecological validity and translational value.

The effects here reported develop our recent evidence of a beneficial cognitive effects of n-3 PUFA supplementation in aged mice (Cutuli et al., 2014) and indicate that the n-3 PUFA-induced hippocampal changes observed at the cellular scale are associated to the macro-scale structural alterations detectable with MRI mapping. Furthermore, the here observed improvements in many facets of mnesic (localizatory, discriminative and social) function, convincingly support an overall n-3 PUFA pro-cognitive action in aging. n-3 PUFA interventional studies in humans also sustain this view evidencing delayed cognitive decline in elderly people with (Yurko-Mauro et al., 2010) or without (Danthiir et al., 2011) subjective memory complaints, and in patients with mild cognitive impairment (Chiu et al., 2008) or very mild AD (Freund-Levi et al., 2006).

Importantly, in the present research n-3 PUFA supplementation exerted beneficial effects not only on cognitive, but also on emotional behaviors. Specifically, n-3 PUFA supplemented mice showed more active coping responses, without inter-group differences in anxiety levels. It is well-known that depression is a multifaceted disorder frequently associated with aging, metabolic disorders and neurodegenerative diseases (Lang and Borgwardt, 2013), and that it is linked to prefrontal and hippocampal atrophy (McNamara and Liu, 2011; Erickson et al., 2012; Vu and Aizenstein, 2013). In agreement with the few previous experimental and clinical findings (Puri et al., 2001; Schipper et al., 2011; Samieri et al., 2012; Lang and Borgwardt, 2013), our results indicate that n-3 PUFA supplementation is able to improve coping abilities by preserving fronto-hippocampal functionality. As a further note, it is important to remember that the n-3 PUFA deficiency has been associated with the dysfunction of neuronal membrane stability and catecholaminergic neurotransmission linked to the etiology of depressive symptoms (Su, 2009). Recently, it has been proposed that EPA and DHA increase serotoninergic transmission by reducing prostaglandin levels and increasing neuronal membrane fluidity (Patrick and Ames, 2015). Given that in the Porsolt test selective serotonin and norepinephrine reuptake inhibitors are reported to increase swimming and climbing behaviors respectively (Renault and Aubert, 2006), we cannot exclude that the n-3 PUFA beneficial effects may be ascribed also to an influence of these nutrients on serotoninergic and noradrenergic neurotransmission.

The use of a three-dimensional hypothesis-independent GM mapping approach allowed us to identify following n-3 PUFA supplementation extra-hippocampal foci of increased GM volume, such as retrosplenial and prefrontal areas. Analogous findings have been recently reported in an interventional study in aged humans receiving prolonged n-3 PUFA supplementation describing improved cognitive functions and increased GM volumes in the hippocampus, precuneus (area reciprocally connected with the adjacent retrosplenial cortex) and frontal areas (Witte et al., 2014). Although the exact mechanisms underlying the involvement of cortical regions remain to be determined, it can be advanced that in n-3 PUFA supplemented mice the preservation of prefrontal structural integrity is functionally driven by the direct afferents stemming from CA1 and subicular hippocampal regions (Hoover and Vertes, 2007). This hypothesis is consistent with enhanced neuroplasticity phenomena (such as increased neurite outgrowth, synaptogenesis, angiogenesis), and decreased neurodegenerative processes (such as apoptosis, astrocytosis) observed in the hippocampus of n-3 PUFA supplemented animals (Gómez-Pinilla, 2008; Thomas and Baker, 2013; Cutuli et al., 2014; Dyall, 2015). Speculatively, it can be hypothesized that the same neuroplastic processes may act at prefrontal and retrosplenial level promoting structural preservation.

Finally, the contribution of WM changes should also be taken into account. Indeed, recent correlational studies reported positive associations between n-3 PUFA levels and GM or WM volumes (Bowman et al., 2012, 2013; Samieri et al., 2012; Tan et al., 2012; Titova et al., 2013; Virtanen et al., 2013; Pottala et al., 2014; Raji et al., 2014). Recently, Witte et al. (2014) suggested that the superior WM microstructural architecture of n-3 PUFA supplemented older adults could be linked to higher myelination, increased fiber packing density and reduced axonal damage that sustain better cognitive performances by improving axonal transmission.

The presence of positive regional association between n-3 PUFA brain levels and GM volumes might be linked to increased regional volume resulting from n-3 PUFA induced increased membrane fluidity and reduced neuroinflammation processes. Specifically, research on the aging brain has shown that major biochemical changes affect the neuronal membrane that is the site of action for many essential functions, such as neurotransmission, regulation of membrane-bound enzymes, control of the ionic channels structure and activity, and receptors maintenance (Yehuda et al., 2002). During aging, the level of cholesterol and its toxic metabolites greatly increases in neuronal membranes, thus reducing the membrane fluidity. On the other hand, n-3 PUFA concentration in aged neuronal membranes decreases (Yehuda, 2012). Interestingly, in the present study EPA and DHA levels, and n-3 PUFA/AA ratio increased following n-3 PUFA supplementation. Thus, it can be argued that by increasing membrane fluidity, n-3 PUFA supplementation may prevent and/or counteract brain aging.

In addition, EPA and DHA have an anti-inflammatory role by giving rise to mediators, such as resolvins and neuroprotectin D1 (Bazan et al., 2011; Calder, 2011), and decreasing age-related microglial activation, oxidative stress, and increased pro-inflammatory cytokines (Martin et al., 2002; Maher et al., 2004; Lynch et al., 2007; Kelly et al., 2011; Trépanier et al., 2015). Accordingly, the present increased n-3 PUFA brain concentrations may result in anti-inflammatory effects, thus contributing to neuroprotective actions against brain atrophy and cognitive decline. Among the multifactorial processes underlying n-3 PUFA beneficial effects on brain structural parameters, cognition, and affective regulation, also theincreased monoaminergic and cholinergic neurotransmission should be taken into account (Willis et al., 2009; Jiang et al., 2012). In any case, future research on this topic is warranted to pinpoint the cellular and sub-cellular determinants of n-3 PUFA induced volumetric enhancement at cortical level.

## Conclusion

Collectively, the present findings suggest that n-3 PUFA supplementation in old age helps maintaining brain functionality by counteracting reductions in GM volume in prefrontal and retrosplenial cortices, as well as in hippocampus. In this respect, n-3 PUFA appear ideal candidates for cognition-enhancing and antidepressant nutritional interventions aimed to promote active and healthy aging. This issue is of growing relevance, given the pressing need to maintain cognitive functions in the elderly Western population, whose life expectancy increasingly rises. Moreover, our study supports the use of VBM measurements in human population as a surrogate mechanistic marker for n-3 PUFA pro-cognitive action in controlled supplementation trials assessing their therapeutic use in the healthy and diseased aged brain.

## Author Contributions

DC, GS, CC, AG, and LP designed research; DC, PC, DL, FF, CN, MP, AGa, and AG performed research; DC, PC, MP, AG and LP analyzed data; DC, MP, AG, and LP wrote the paper.

## Conflict of Interest Statement

The authors declare that the research was conducted in the absence of any commercial or financial relationships that could be construed as a potential conflict of interest.

## Supplementary Material

**FIGURE S1 | Anatomical location of the hippocampal bilateral cubic ROIs.** Three-dimensional rendering and slice representation of the anatomical location of the bilateral cubic (9 × 9 × 9 voxels) ROIs centered in hippocampal foci exhibiting significant correlations with behavioral scores and PUFA (*p* < 0.01, cluster-based correction at a significance level of 0.01). HPC, hippocampus.

**FIGURE S2 | Tensor Based Morphometry (TBM) corroborates supplementation-specificity of increased GM volume mapped with VBM.** n-3 PUFA supplementation increases hippocampal and prefrontal GM volume as seen with TBM (*p* < 0.01, cluster-based correction at a significance level of 0.01). Note the overlap with VBM analysis (**Figure 4**). HPC, hippocampus; mPFC, medial prefrontal cortex; RS, retrosplenial cortex; OFC, orbitofrontal cortex.

**FIGURE S3 | Behavioral performances are positively correlated with fronto-hippocampal GM volume.** Consistent with univariate inter-group mapping, voxel-wise correlation of behavioral scores and GM volume prior to cluster-based correction revealed foci of significant correlation in hippocampal and prefrontal regions. GM-behavioral performance correlation map for MWM spatial mnesic performances **(A)** and coping skills in the Porsolt test **(B)**. The prefrontal effects did not survive cluster-based correction (*p* = 0.01, **Figure 5**). HPC, hippocampus; mPFC, medial prefrontal cortex; RS, retrosplenial cortex.

**FIGURE S4 | Positive correlations between n-3 PUFA levels and fronto-hippocampal GM volume.** Consistent with univariate inter-group mapping, voxel-wise correlation between total brain EPA **(A)**, DHA **(B)** and n-3 PUFA/AA concentrations **(C)** and GM volume prior to cluster-based correction revealed foci of significant correlation in hippocampal and prefrontal regions. For EPA, prefrontal effects did not survive cluster-based correction (*p* = 0.01, **Figure 6**). EPA, eicosapentaenoic acid; DHA, docosahexaenoic acid; AA, arachidonic acid; HPC, hippocampus; OFC, orbitofrontal cortex; mPFC, medial prefrontal cortex; RS, retrosplenial cortex.
