# Supplementary figures and images for: Effects of Omega-3 Fatty Acid Supplementation on Cognitive Functions and Neural Substrates: A Voxel-Based Morphometry Study in Aged Mice
Source: Front Aging Neurosci. 2016 Mar 4;8:38. doi: 10.3389/fnagi.2016.00038 (PMC4777728; doi:10.3389/fnagi.2016.00038)

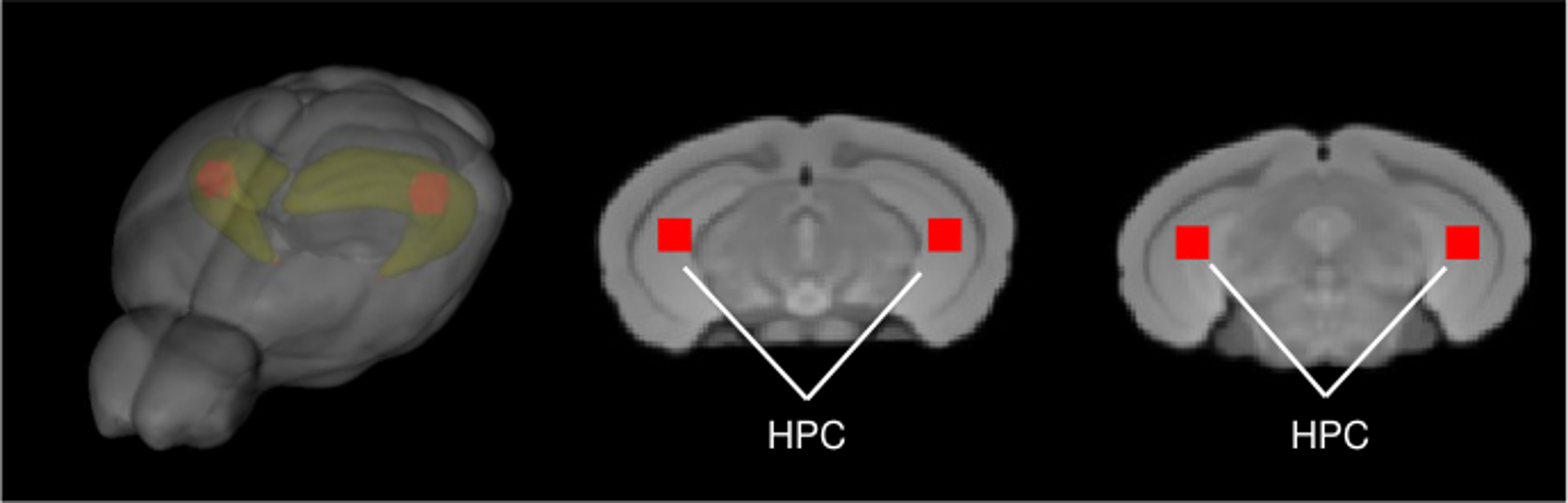

Supplement: Supplementary file 1 [file Image_1.TIF]

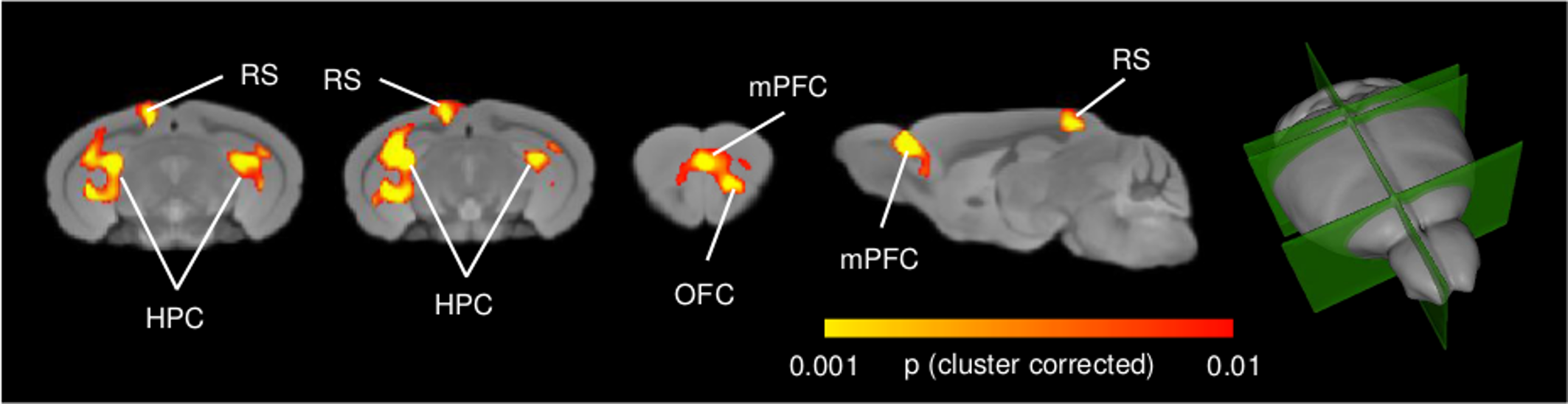

Supplement: Supplementary file 2 [file Image_2.TIF]

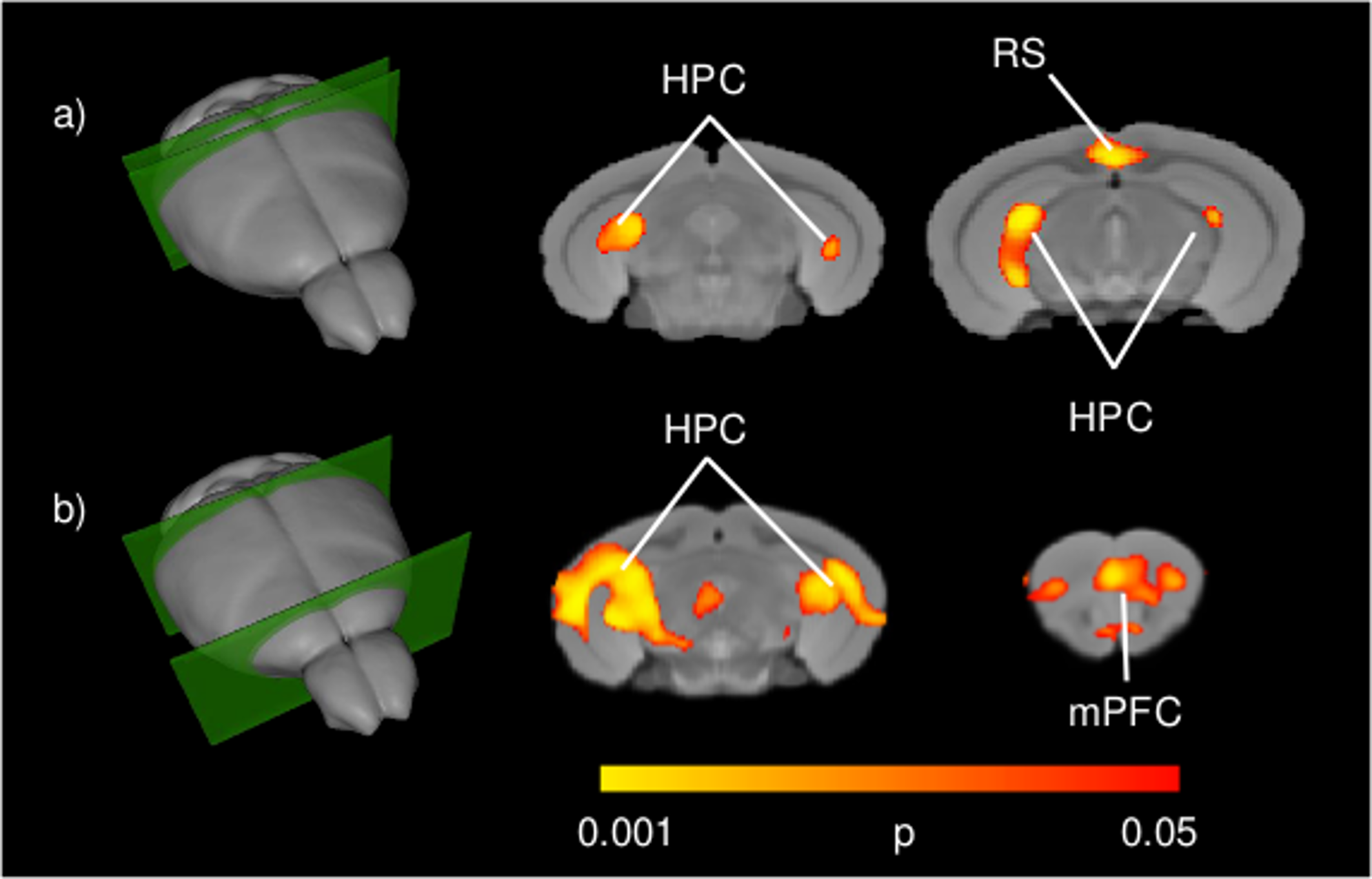

Supplement: Supplementary file 3 [file Image_3.TIF]

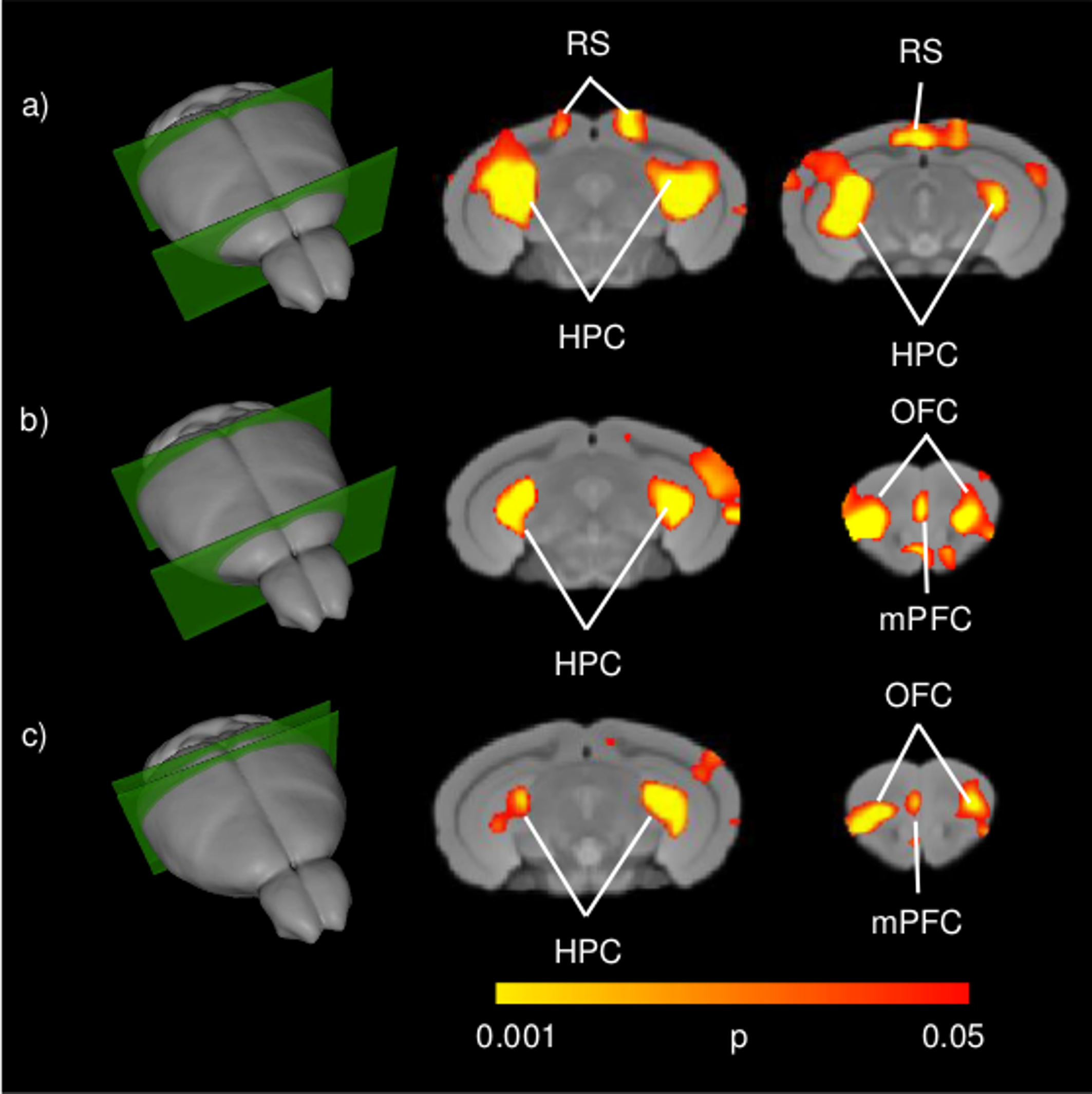

Supplement: Supplementary file 4 [file Image_4.TIF]
